# Exploring Bacteria from Extreme Environments in Chilean Patagonia for Antitrypanosomal Metabolites

**DOI:** 10.1021/acsomega.6c07469

**Published:** 2026-10-13

**Authors:** Vanessa Albuquerque, Julia Maria Godoi Lima, Marina de Monroe Gonçalves, Jose Aparecido Ferreira de Lima, Ravi Kant, Myron Christodoulides, Daniel Carvalho Pimenta, João Henrique Ghilardi Lago, Cledir Santos, Andre Gustavo Tempone

**Affiliations:** † Pathophysiology Laboratory, Instituto Butantan, Av. Vital 1500, São Paulo 05503-900, SP, Brazil; ‡ Centre of Natural Sciences and Humanities, Federal University of the ABC, São Paulo 09210-580, SP, Brazil; § Laboratory of Ecology & Evolution, Instituto Butantan, Av. Vital Brazil, 1500, São Paulo 05503-900, SP, Brazil; ∥ Computational Drug & Vaccine Discovery Laboratory, Faculty of Applied Sciences & Biotechnology, 375605 Shoolini University, Solan, Himachal Pradesh 173229, India; ⊥ Molecular Microbiology, School of Clinical and Experimental Sciences, Faculty of Medicine, 7423 University of Southampton, Southampton SO16 6YD, England, U.K.; # Scientific and Technological Bioresource Nucleus (BIOREN-UFRO), 664812 Universidad de La Frontera, Temuco 4811-230, Chile; ∇ Postgraduate Program in Biotechnology, Federal University of Technology, Ponta Grossa, Paraná 84017220, Brazil

## Abstract

Extreme environments are recognized as valuable reservoirs of microbial diversity and chemical novelty. Adapted to harsh environmental conditions, bacteria isolated from extreme environments exhibit unique biosynthetic capabilities associated with the production of structurally diverse bioactive metabolites. Chagas disease (CD), caused by the protozoan parasite *Trypanosoma cruzi*, remains one of the most important neglected tropical diseases, affecting approximately eight million people worldwide. Current chemotherapy relies on only two drugs, benznidazole and nifurtimox, both associated with toxicity and therapeutic limitations. The search for new antitrypanosomal agents has therefore intensified, directing attention to underexplored microbial resources such as bacteria isolated from extreme environments, which are known for their remarkable biosynthetic potential and chemical diversity. In this context, the present study investigated the anti-*T. cruzi* potential of extracts obtained from sediment-associated microbial communities from the Magallanes Region of Chilean Patagonia. By using 16S rRNA sequencing, seven bacterial isolates from extreme environments were identified as belonging to the genus *Roseovarius* spp., *Loktanella* spp., *Pseudarthrobacter* spp., *Pseudomonas* spp., *Psychroflexus* spp., and *Cyclobacterium* spp. The antiparasitic activity of the extracts was determined in trypomastigotes, resulting in potent IC_50_ values ranging from 2 to 18 μg/mL. Bioactivity-guided fractionation of the *Cyclobacterium plantarum* extract led to the putative identification of VAS8, a phosphatidylethanolamine derivative, as its active constituent. VAS8 exhibited activity against both trypomastigote (IC_50_ = 49 μM) and intracellular amastigote (IC_50_ = 19 μM) forms of *T. cruzi*, while showing no significant cytotoxicity toward mammalian host cells (CC_50_ > 150 μM). Spectrofluorimetric and fluorescence microscopy studies using SYTOX Green demonstrated a time/concentration-dependent permeabilization of the plasma membrane after VAS8 incubation with trypomastigotes. These findings highlight bacteria isolated from extreme environments as a promising source of antitrypanosomal metabolites and phosphatidylethanolamines as attractive scaffolds for the development of new therapies against Chagas disease.

## Graphical abstract

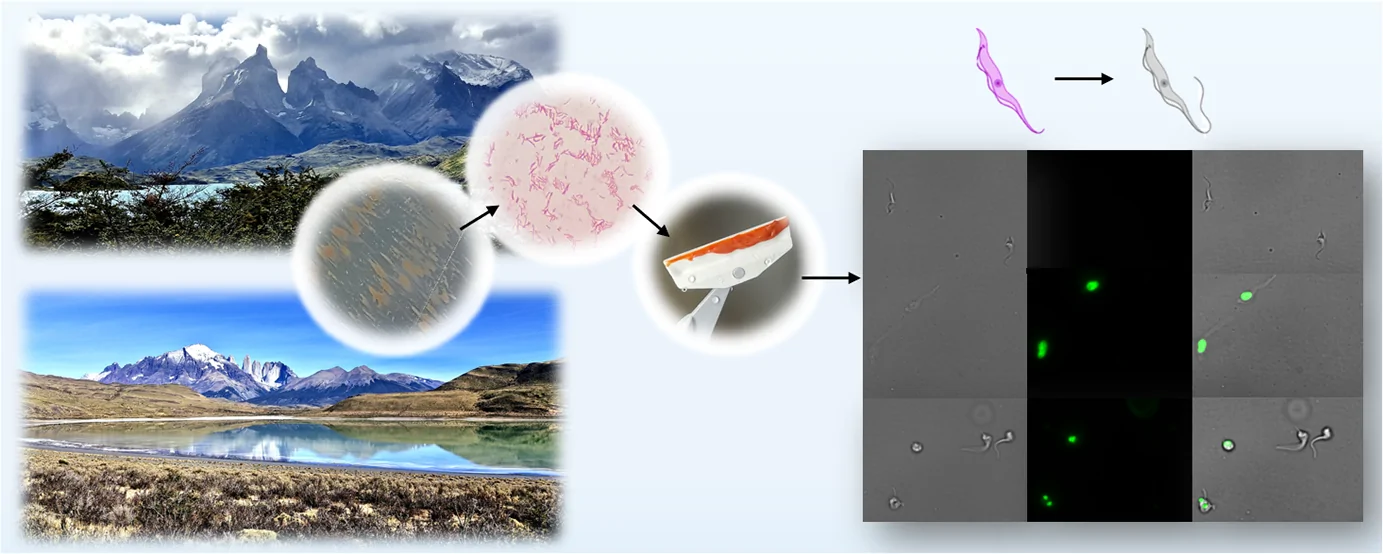

## 1 Introduction

Extreme environments are characterized by physicochemical, geological, or nutritional conditions that challenge the limits of biological viability.^1^ These habitats encompass a wide range of ecosystems, including hypersaline lakes, glacial environments, hydrothermal systems, and polar regions, where strong selective pressures shape the structure and metabolic potential of resident microbial communities.^2^ To survive under these harsh conditions, bacteria isolated from extreme environments have evolved a wide range of cellular, biochemical, and molecular adaptations that ensure metabolic functionality and ecological fitness.^3^ Among these adaptations is the production of metabolites, low-molecular-weight compounds that play essential roles in microbial defense, competition, and communication.^4^ Owing to their unique evolutionary histories and biosynthetic potential, bacteria isolated from extreme environments have emerged as prolific sources of structurally diverse bioactive metabolites, attracting increasing attention in natural product–based drug discovery efforts.^5^ Among the therapeutic areas that may benefit from the exploration of these underinvestigated microbial resources, Chagas disease remains a compelling target due to the limited efficacy and toxicity associated with current treatments. Chagas disease, caused by the protozoan parasite *Trypanosoma cruzi*, affects approximately 8 million people worldwide and remains a major public health challenge Although historically endemic to Latin America, increasing human migration has expanded its geographic distribution, raising concern in nonendemic regions.^6^ The disease follows a biphasic course, consisting of an acute stage characterized by high parasitemia and the predominance of infective bloodstream trypomastigotes, and a chronic stage characterized by the long-term persistence and intracellular replication of amastigotes within host tissues. Approximately 30–40% of chronically infected individuals develop severe cardiac or gastrointestinal complications, which account for most disease-associated morbidity and mortality.^7^ Despite its considerable public health burden, chemotherapy remains restricted to only two drugs, benznidazole and nifurtimox, both associated with prolonged treatment regimens, severe adverse effects, and limited efficacy during chronic infection, that compromise patient adherence.^8^ These limitations underscore the urgent need for novel antitrypanosomal agents with improved safety profiles and therapeutic efficacy. In this context, increasing attention has been directed toward underexplored microbial resources with unique biosynthetic capabilities as potential sources of new antiparasitic compounds.^9^ Accordingly, the present study investigated sediment-associated bacteria isolated from extreme environments from the Magallanes Region of Chilean Patagonia as a source of bioactive metabolites active against *T. cruzi*.

## 2 Results

### 2.1 Samples Collection, Isolation and Identification of Associated Bacteria Isolated from Extreme Environments

Seven cultivable bacterial isolates were obtained from sediment samples collected in Laguna Amarga (Bitter Lagoon) and Lago Grey (Gray Lake), two contrasting extreme environments located in the Magallanes Region of Chilean Patagonia. Taxonomic identification based on partial 16S rRNA gene sequencing and comparison with the Genome Taxonomy Database (GTDB) assigned the isolates to the species *Cyclobacterium plantarum*, *Loktanella agnita* *, Psychroflexus* sp., *Roseovarius gahaiensis*, and *Roseovarius halotolerans*, from the Bitter Lagoon sediment, and *Pseudarthrobacter humi* and *Pseudomonas baetica* from the Gray Lake sediment. The percentage identities obtained for each isolate are summarized in Table 1, while sequence alignments are provided in Supporting Information 1.

**Table 1:** Identification of Marine Bacteria by the 16S rRNA Gene, Using the GTBD^a^

Acronym	Identification	Identity/%
PLASE 1	*Cyclobacterium plantarum*	99
PLASE 2	*Loktanella agnita*	100
PLGSE 1	*Pseudarthrobacter humi*	99
PLGSE 2	*Pseudomonas baetica*	100
PLASE 3	*Psychroflexus* sp.	96
PLASE 4	*Roseovarius gahaiensis*	99
PLASE 5	*Roseovarius halotolerans*	99

a Abbreviations: PLASE—Patagonian Sediments from Bitter Lagoon; PLGSE—Patagonian Sediments from Gray Lake; GTDB—Genome Taxonomy Database.

### 2.2 Preparation and Evaluation of the Antiparasitic Activity of Extracts

Following taxonomic identification, all bacterial isolates were cultivated for metabolite production and were subjected to extraction procedures, as indicated in Table 2.

**Table 2:** Mass of Extracts of the Isolated Bacteria Isolated from Extreme Environments

Bacterium	Mass/mg
*Cyclobacterium plantarum*	58.9
*Loktanella agnita*	16.4
*Pseudarthrobacter humi*	49.2
*Pseudomonas baetica*	26.5
*Psychroflexus* sp.	24.3
*Roseovarius gahaiensis*	22.3
*Roseovarius halotolerans*	18.5

The antiparasitic activity of the extracts was evaluated against trypomastigotes of *T. cruzi*. All extracts exhibited activity against the parasite and completely eliminated trypomastigotes at the highest tested concentration (150 μg/mL) (Figure 1), with IC_50_ values ranging from 2 to 18 μg/mL (Table 3). The most potent extracts were obtained from Roseovarius halotolerans and *Roseovarius gahaiensis*, with IC_50_ values of 2.7 and 4.0 μg/mL, respectively. Notably, both strains belong to the same bacterial genus, whereas the remaining isolates displayed IC_50_ values distributed throughout the evaluated range.

**Figure 1:**
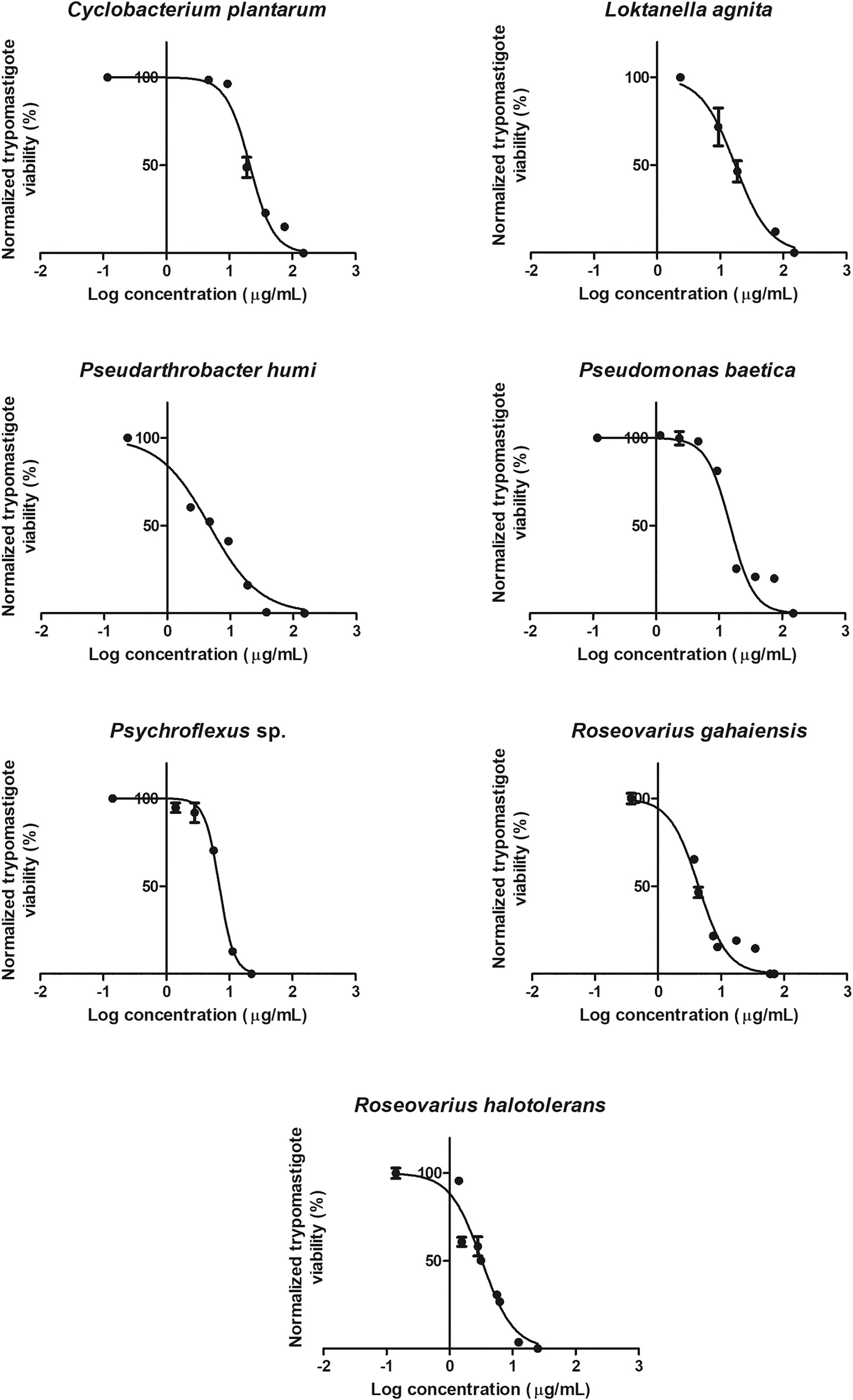
Concentration–response curve graphs of bacterial extracts against trypomastigotes of *T. cruzi*.

**Table 3:** Antiparasitic Activity (Trypomastigotes of *T. cruzi*) of Metabolites from Bacteria Isolated from Extreme Environments Isolated from Sediments^a^

Bacterium	IC_50_ ± S.D./μg/mL
*Cyclobacterium plantarum*	18.6 ± 3.4
*Loktanella agnita*	15.5 ± 2.5
*Pseudarthrobacter humi*	4.7 ± 0.6
*Pseudomonas baetica*	13.0 ± 2.8
*Psychroflexus* sp.	7.7 ± 0.6
*Roseovarius gahaiensis*	4.0 ± 0.5
*Roseovarius halotolerans*	2.7 ± 0.7

a Abbreviations: IC_50_—50% Inhibitory Concentration; SD—Standard Deviation.

### 2.3 Bioactivity-Guided Fractionation of *Cyclobacterium plantarum* Extract

The species *Cyclobacterium plantarum* (Figure 2) was selected for further investigation based on its antitrypanosomal activity and the amount of extract obtained. The ability to produce substantial amounts of extractable metabolites, together with its unexplored biosynthetic potential, supported its prioritization for bioassay-guided fractionation.

**Figure 2:**
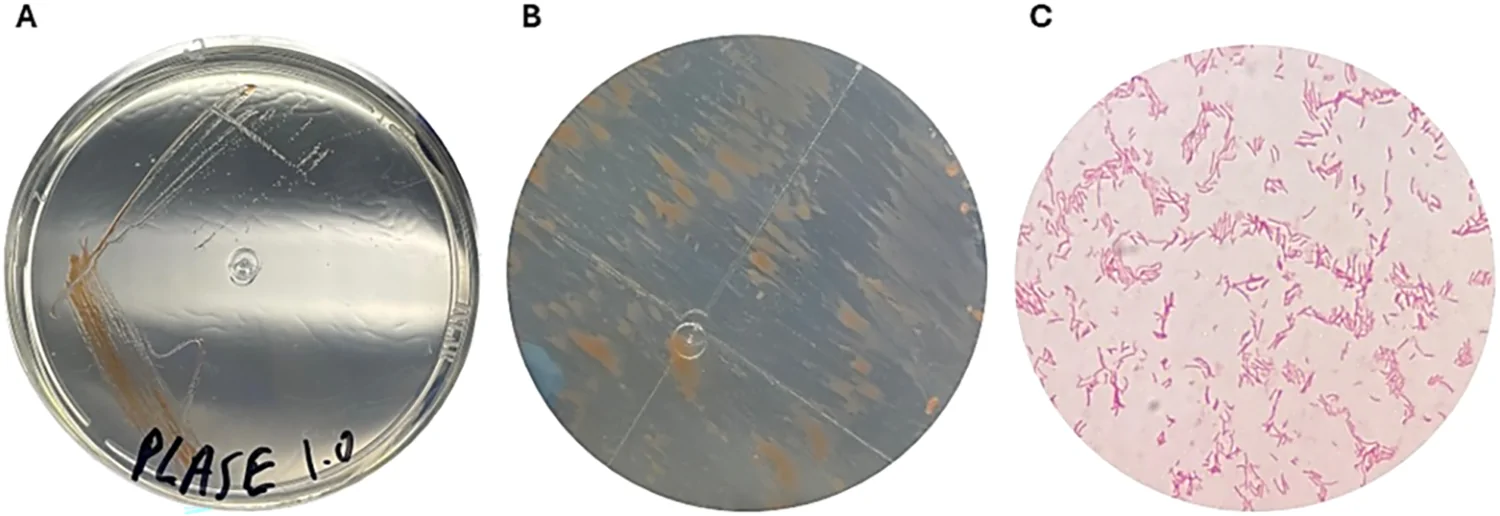
Macroscopic images and micrographs of *C. plantarum*. (A) – Colonies of the isolate in marine agar. (B) – Petri plate (150 × 90 mm) image showing confluent bacterial growth for upscaling. (C) – Gram staining of the isolate, revealing morphotintorial characteristics consistent with Gram-negative bacilli.

The extract from *C. plantarum* was successively partitioned using hexane and EtOAc. The bioactive EtOAc phase was subjected to a reverse phase (C_18_) chromatographic fractionation to afford three fractions (F1–F3). Fraction F2 exhibited activity and was selected for purification using C_18_ semipreparative HPLC to afford compound VAS8 as an amorphous white solid (97% of purity by HPLC). The ESI-HRMS (positive mode—Figure S1) data analysis showed the signals of [M + H]^+^ and [M + Na]^+^ ions at *m*/*z* 731.5373 and 753.5245, respectively, in accordance with molecular formula C_40_H_77_NO_8_P to VAS8. Additionally, the diagnostic ion at *m*/*z* 590.4 [M + H – 141]^+^, corresponding to the loss of the phosphoethanolamine headgroup, together with the ions at *m*/*z* 253.3 and 265.2, corresponding to acyl group fragments, were observed in the MS/MS spectrum (Figures S2–S5). Based on its accurate mass and characteristic fragmentation pattern, compound VAS8 was putatively annotated as shown in Figure 3.

**Figure 3:**
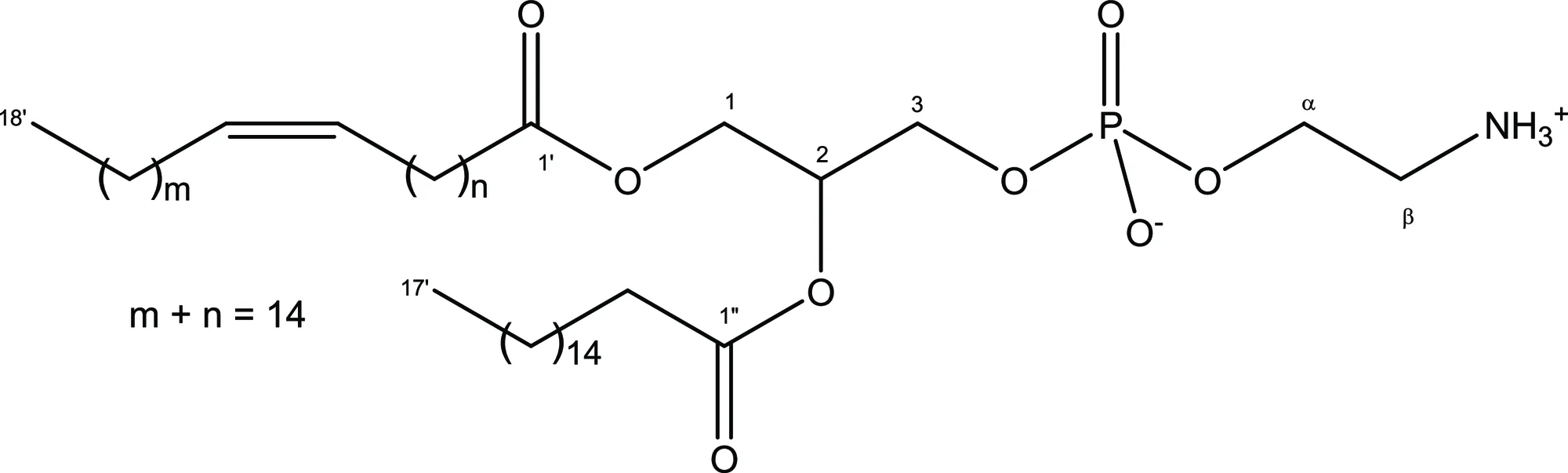
Suggested structure of VAS8, a phosphatidylethanolamine isolated from *C. plantarum*, based on mass spectrometric data. It is important to note that the acyl side chains may be interchanged between positions C-1 and C-2.

The activity observed in fraction F2 suggests that compounds other than VAS8 may also contribute to the anti-*T. cruzi* activity. However, the amounts obtained from the other subfractions were insufficient to allow further biological evaluation or complete structural elucidation. Therefore, the subsequent biological and chemical investigations focused on VAS8.

### 2.4 Antitrypanosomal Activity of VAS8 and Phenotypic Evaluation of *T. cruzi* Plasma Membrane Permeability

The antitrypanosomal activity of VAS8 demonstrated that the compound eliminated 100% of *T. cruzi* trypomastigotes, with an IC_50_ value of 49.5 ± 3.4 μM. In comparison, benznidazole, the standard drug used for the treatment of Chagas disease, exhibited an IC_50_ value of 15.7 ± 0.6 μM. The purified compound VAS8 was less potent than the original crude extract when its IC_50_ was expressed in mass concentration, suggesting additional active constituents. Although other fractions were obtained during bioassay-guided fractionation, their recovered amounts were insufficient for biological evaluation and structural elucidation. In intracellular amastigote assays (Figure 4), VAS8 reduced both the number of parasites per macrophage and the percentage of infected macrophages at 100 μM while preserving host-cell morphology, resulting in an IC_50_ of 19.5 ± 1.9 μM. Under the same conditions, benznidazole displayed an IC_50_ of 3.9 ± 0.3 μM. Cytotoxicity assays (CC_50_) against murine peritoneal macrophages revealed CC_50_ higher than 150 μM for VAS8, corresponding to a selectivity index (SI) > 7.7. Benznidazole showed a CC_50_ higher than 150 μM and an SI > 38.4. The biological activity, cytotoxicity, and selectivity index values are presented in Table 4.

**Figure 4:**
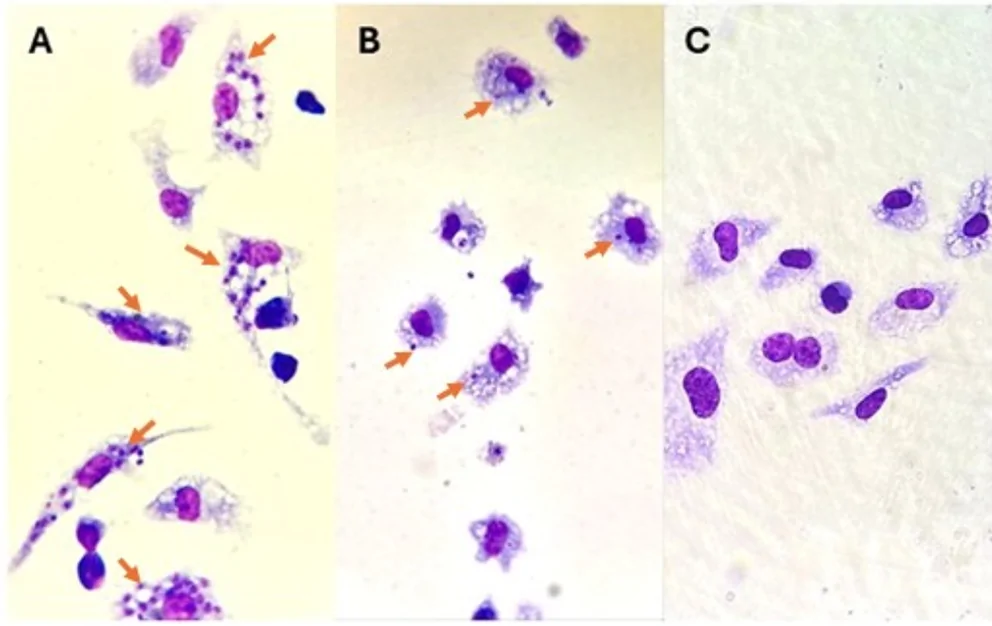
Representative light microscopy images of intracellular *T. cruzi* amastigotes in infected macrophages. (A) Untreated control; (B) VAS8-treated cells (100 μM); and (C) benznidazole-treated cells (100 μM). Orange arrow – intracellular amastigotes. Images were acquired at 100 × magnification.

**Table 4:** Antitrypanosomal Activity of Compound VAS8 against *T. cruzi* Trypomastigotes and Intracellular Amastigotes, Cytotoxicity toward Murine Peritoneal Macrophages, and Corresponding Selectivity Index (SI) Values^a^

Compound	IC_50_ ± SD trypomastigotes/μM	IC_50_ ± S.D. amastigotes/μM	CC_50_murine peritoneal macrophages/μM	SI
VAS8	49.5 ± 3.4	19.5 ± 1.9	>150	>7.7
Benznidazole	15.7 ± 0.6	3.9 ± 0.3	>150	>38.4

a Abbreviations: IC_50_ – 50% Inhibitory Concentration; SD – Standard Deviation.

To gain initial insights into its cellular effects, the activity of VAS8 on *T. cruzi* plasma membrane permeability was evaluated. The results shown in Figure 5 indicate that, compared with the untreated control, VAS8 significantly (*p* < 0.05) altered the plasma membrane permeability of *T. cruzi* at all tested concentrations (IC_50_ and 2 × IC_50_) and time points evaluated. VAS8 induced a significant increase in fluorescence levels at both concentrations during the early incubation periods. However, at 2 × −IC_50_, the compound produced the highest fluorescence levels, comparable to those observed for the positive control treated with the detergent Triton X-100.

**Figure 5:**
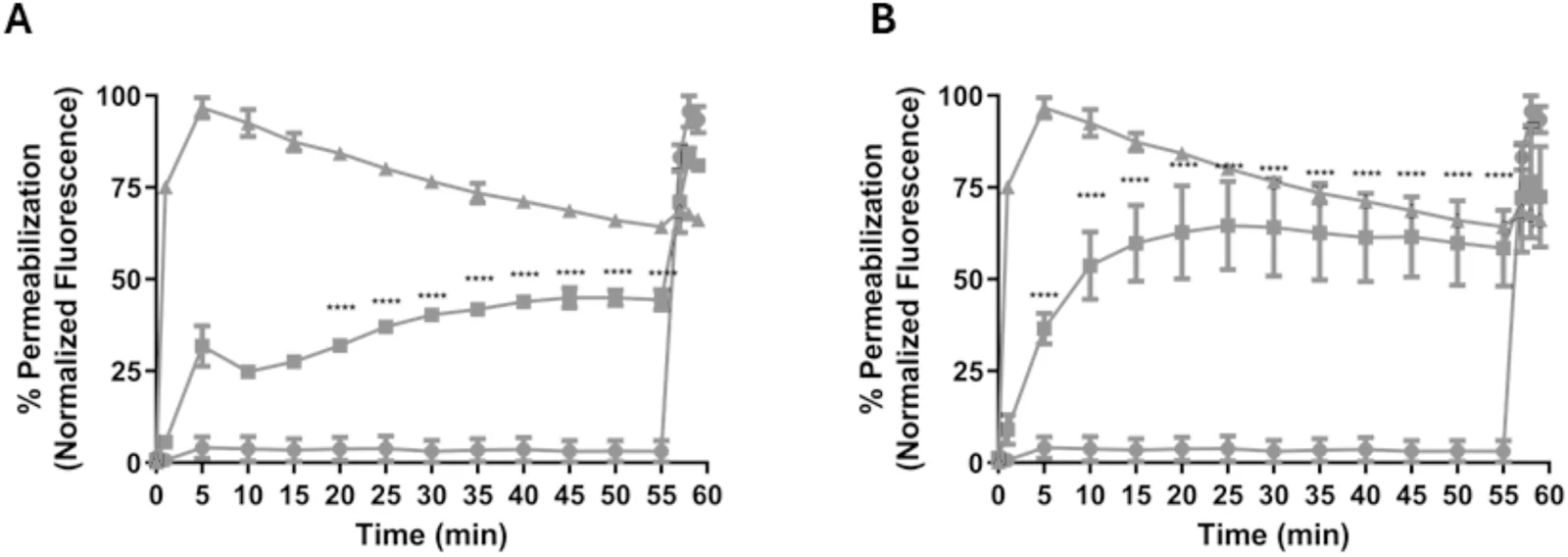
Evaluation of *T. cruzi* trypomastigotes plasma membrane permeability using the fluorescent probe SYTOX Green and spectrofluorimetric measurements (excitation, λ = 504 nm; emission, λ = 523 nm). Circles, untreated parasites (negative control), squares, parasites treated with VAS8, and triangles, parasites treated with Triton X-100 (positive control). (A) Parasites treated with VAS8 at the previously determined IC_50_; (B) parasites treated with VAS8 at 2 × −IC_50_. Data are presented as mean ± standard deviation of technical triplicates from a representative experiment. Statistical analysis was performed using two-way ANOVA followed by Šídák’s multiple comparisons test. ****p < 0.0001 compared with the untreated control.

To validate the spectrofluorimetric findings obtained with the SYTOX Green assay, fluorescence microscopy images of *T. cruzi* trypomastigotes were acquired following 30 min of exposure to VAS8, a time point at which both tested concentrations significantly increased plasma membrane permeabilization relative to the untreated control. The results shown in Figure 6A3, demonstrate the merged image of untreated trypomastigotes, with normal elongated morphology and no fluorescence in the intracellular millie, confirming the nonaltered plasma membrane. As expected, Triton X-100 induced intense fluorescence labeling (nucleus) and served as the positive control for plasma membrane disruption (Figure 6B3). In agreement with the quantitative fluorescence data (spectrofluorimetric analysis), parasites treated with VAS8 at the IC_50_ and 2 × −IC_50_ displayed markedly higher fluorescence signals (Figure 6C3) than untreated parasites, and an intense altered morphology (round form), indicating enhanced membrane permeability.

**Figure 6:**
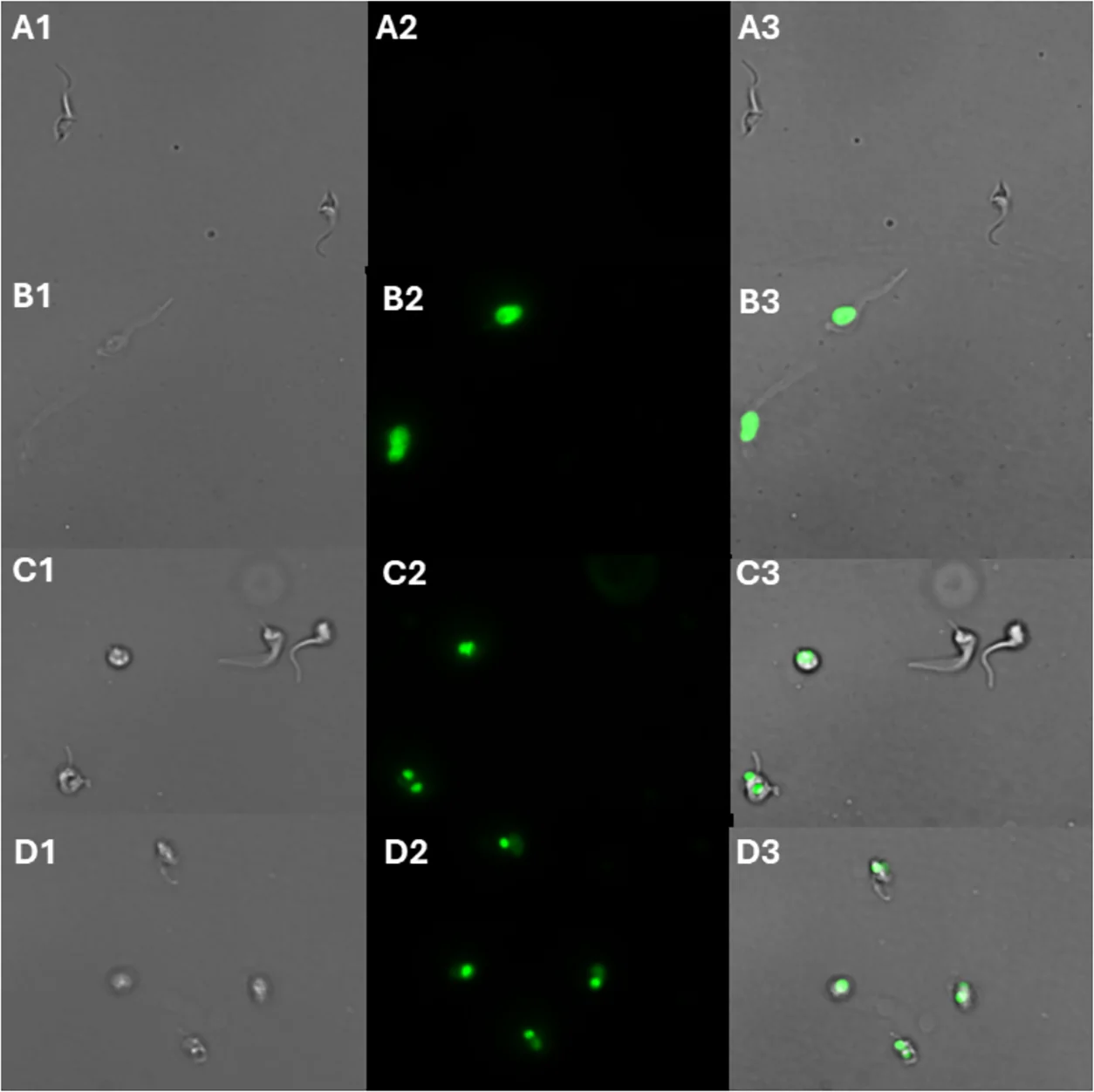
Representative fluorescence microscopy images of *T. cruzi* trypomastigotes stained with SYTOX Green following different treatments. (A) Untreated control; (B) Triton X-100 (positive control); (C) VAS8 at the previously determined IC_50;_ and (D) VAS8 at 2 × −IC_50_. Columns show bright-field images (1), fluorescence images (2), and merged images (3). Images were acquired using an EVOS M5000 digital microscope (Thermo Fisher Scientific, USA). Black bar corresponds to 15 μm.

## 3 Discussion

The biological complexity of Chagas disease and the limited efficacy of current therapeutic options continue to drive the search for new anti-*T. cruzi* agents.^10^ In this regard, bacteria isolated from extreme environments have attracted increasing biotechnological interest due to their ability to thrive under harsh physicochemical conditions, a feature often associated with the production of structurally distinct metabolites. Consequently, extreme environments represent promising and still underexplored sources of microbial and chemical diversity, offering valuable opportunities for the discovery of novel bioactive compounds.^11,9^

In the present study, species belonging to the genus *Roseovarius* significantly expanded the pharmacological knowledge associated with bacteria adapted to extreme environments. *Roseovarius gahaiensis*, first described in 2020 from a Chinese saline lake,^12^ exhibited anti-*T. cruzi* activity that had not previously been reported for the species. Similarly, *Roseovarius halotolerans*, a halophilic bacterium that requires high salt concentrations for growth,^13^ not only displayed previously unreported anti-*T. cruzi* activity but also yielded the most potent antiparasitic extract among the bacteria isolated from extreme environments evaluated. Although the genus *Roseovarius* has been associated with antimicrobial activity,^14^ to the best of our knowledge, this study provides the first evidence of its anti-*T. cruzi* potential.

Species belonging to the genera *Psychroflexus*, *Loktanella*, and *Pseudarthrobacter* also expanded the known pharmacological repertoire of bacteria isolated from extreme environments. *Psychroflexus* sp., previously described in Chinese marsh environments,^15^ exhibited potent antiparasitic activity, representing, to the best of our knowledge, the first report of biological activity for the genus *Psychroflexus*. Likewise, Loktanella agnita, a species predominantly associated with marine and extreme environments,^16,17^ displayed previously unreported anti-*T. cruzi* activity, expanding the biotechnological potential previously attributed to the genus, whose known activities were mainly related to algicidal effects.^18^ *Pseudarthrobacter humi*, recently described from soils of the Republic of Korea,^19^ belongs to a genus frequently isolated from cold and glacial environments.^20,21^ In the present study, the species exhibited antiparasitic activity not previously reported for the genus *Pseudarthrobacter*, which had been primarily associated with antimicrobial activity.^22^

Consistent with the findings for the other bacteria isolated from extreme environments evaluated, *Pseudomonas baetica* exhibited anti-*T. cruzi* activity that had not previously been described for the species. Although it has been associated with antifungal activities,^23^ to the best of our knowledge, there are no previous reports of its antiparasitic potential. These findings expand the spectrum of biological activities attributed to the species and corroborate the anti-*T. cruzi* potential previously reported for the genus *Pseudomonas*.^24^

*Cyclobacterium plantarum*, a bacterium isolated from extreme environment described only recently,^25^ was selected for further investigation because of its high extract yield and antitrypanosomal potency. Although its extract did not exhibit the highest antitrypanosomal activity among the bacteria isolated from extreme environments evaluated, the combination of substantial metabolite production and the limited knowledge available on the genus *Cyclobacterium* justified its selection for bioactivity-guided fractionation. This approach led to the isolation of VAS8, which was putatively identified as a phosphatidylethanolamine derivative and displayed activity against both trypomastigote and intracellular amastigote forms of *T. cruzi*, while exhibiting low cytotoxicity toward mammalian cells. In addition, light microscopy analyses revealed a marked reduction in the number of intracellular parasites within the host cell cytoplasm following treatment. Activity against intracellular amastigotes is of particular pharmacological relevance, as this replicative stage is responsible for parasite persistence in the vertebrate host and represents one of the major therapeutic challenges in Chagas disease.^26^ Furthermore, according to Drugs for Neglected Diseases *initiative* (DND*i*) guidelines, compounds acting exclusively against replicative forms may allow nonreplicative parasites, such as trypomastigotes, to sustain infection after treatment cessation, highlighting the importance of identifying hit compounds active against both clinically relevant stages of the parasite,^27^ as observed for VAS8. Although the IC_50_ value against amastigotes and the selectivity index did not meet the ideal hit criteria established by DND*i* for Chagas disease (IC_50_ < 10 μM and SI > 10),^27^ VAS8 remains within a favorable therapeutic window, considering that future medicinal chemistry efforts may improve both potency and selectivity.

Phosphatidylethanolamines constitute one of the major classes of glycerophospholipids found in mammalian cell membranes, playing essential roles in maintaining the structural organization of the lipid bilayer, modulating membrane fluidity and curvature, and regulating protein–membrane interactions.^28^ Variations in acyl chain length and degree of unsaturation contribute to the structural diversity of bacterial phospholipids and may reflect differences in lipid metabolism among organisms. In *T. cruzi*, phosphatidylethanolamines are among the phospholipid constituents of cellular membranes and participate in processes related to bilayer organization.^29^ Given that lipid biosynthesis, remodeling, and utilization are essential for parasite survival and metabolic adaptation, lipid metabolism has emerged as an increasingly attractive source of potential therapeutic targets.^29^

Previous studies have demonstrated that *T. cruzi* can acquire host-derived lipids and incorporate them into its own lipidome, complementing *de novo* biosynthesis and fatty acid remodeling processes.^30^ In this context, the structural similarity between VAS8 and phosphatidylethanolamines naturally present in the parasite, suggests that this compound may interact with components of the parasite plasma membrane, thereby contributing to the disruption of lipid homeostasis.

To investigate whether this interaction could be associated with alterations in the parasite plasma membrane, permeability assays using the fluorescent probe SYTOX Green were performed. VAS8-treated trypomastigotes exhibited a progressive increase in fluorescence, indicating that changes in plasma membrane permeability were dependent on exposure time. This effect was more pronounced at 2 × −IC_50_, revealing a concentration-dependent response and suggesting that higher concentrations intensified this process. The rapid onset of membrane permeabilization is consistent with an early membrane-directed effect of VAS8; however, additional studies are required to determine whether the plasma membrane is its primary target or whether the observed permeabilization is a secondary consequence of another rapidly initiated cellular event.

Fluorescence microscopy corroborated the spectrofluorimetric findings and provided additional evidence that these events were accompanied by structural alterations in the parasite. Compared with trypomastigotes treated at the IC_50_, parasites exposed to 2 × −IC_50_ exhibited more pronounced morphological changes, including loss of the characteristic elongated morphology and a tendency toward amastigote-like (rounding cells), whereas SYTOX Green-negative parasites retained the typical appearance of viable forms. Together, these observations suggest that the permeability changes induced by VAS8 in trypomastigotes are associated with progressive cellular damage, the intensity of which increases with both compound concentration and exposure time. The ability of exogenous molecules structurally related to endogenous parasite components to modulate *T. cruzi* plasma membrane stability has been previously demonstrated. Alexandre et al.,^31^ showed that incubation of trypomastigotes with ergosterol isolated from a basidiomycete promoted significant alterations in plasma membrane permeability, ultimately leading to parasite death.

The effects of amphipathic molecules on biological membranes have also been described in *T. cruzi*. Although amphotericin B is widely used in the treatment of fungal infections and visceral leishmaniasis,^32,33^ its activity has also been demonstrated against different developmental stages of *T. cruzi*. De Castro et al.^34^ reported ultrastructural alterations in trypomastigotes following exposure to the drug. More recently, Martínez et al.,^35^ demonstrated that the synthetic derivative A21, obtained from amphotericin B, exhibited higher anti-*T. cruzi* activity than the parent compound, significantly reducing parasite number and motility while inducing evident morphological alterations. Although amphotericin B and VAS8 belong to distinct chemical classes, both are amphipathic molecules and may share similarities in their mode of action. In *Leishmania donovani* promastigotes, Saha et al.^36^ demonstrated that amphotericin B disrupts the cellular permeability barrier following interaction with membrane sterols, leading to the leakage of essential intracellular metabolites. Although the findings of the present study do not allow us to infer that VAS8 shares the same pore-forming mechanism described for amphotericin B, the results obtained from permeability assays and fluorescence microscopy support the hypothesis that alterations in plasma membrane permeability may contribute to the trypanocide activity of the compound.

Due to their structural diversity and ability to interact with biologically relevant targets, natural products are frequently employed as prototypes in medicinal chemistry to generate compounds with improved pharmacological properties.^37^ In this context, phosphatidylethanolamines have emerged as promising scaffolds for pharmacological optimization. Balakrishna et al.^38^ demonstrated that modifications to the polar headgroup of a natural phosphatidylethanolamine yielded derivatives active against different human tumor cell lines. Similarly, Vijeetha et al.^39^ obtained phosphatidylethanolamine-N-amino acid derivatives displaying remarkable antimicrobial activity and moderate antifungal effects, demonstrating that structural modifications within this class of lipids can result in substantial changes in their biological profile. Collectively, these findings highlight phosphatidylethanolamines as versatile prototypes amenable to structural modulation and exploration across different therapeutic settings.

This versatility parallels other successful examples of drug development from lipid-derived molecules. Fingolimod, currently used for the treatment of relapsing multiple sclerosis, originated from structure–activity relationship studies involving myriocin, a highly potent microbial metabolite associated with significant *in vivo* toxicity.^40^ Structural simplification of this compound ultimately resulted in a drug with a pharmacological and safety profile compatible with clinical use.^41^ Likewise, miltefosine was originally developed as a synthetic alkylphospholipid structurally related to biological phospholipids for antitumor purposes. Despite the initial interest in this indication, the compound failed to demonstrate satisfactory clinical efficacy for the oral treatment of cancer. The subsequent identification of antiparasitic activity against *Leishmania* spp. species led to its therapeutic repositioning, culminating in the approval of the first oral treatment for visceral leishmaniasis.^42^ These examples illustrate how structural optimization and therapeutic repositioning can transform lipid-based molecules from interesting pharmacological prototypes into clinically relevant candidates. Together, these studies support VAS8 as a candidate for subsequent efforts aimed at the development of structurally and pharmacologically optimized derivatives.

## 4 Conclusions

The present study reports the isolation of VAS8, putatively identified as a phosphatidylethanolamine derivative, from *Cyclobacterium plantarum* isolated from an extreme environment and highlights its potential anti-*Trypanosoma cruzi* activity. Beyond its antiparasitic activity, the identification of a phosphatidylethanolamine with anti-*T. cruzi* potential expands the current understanding of the pharmacological relevance of lipid metabolites produced by microorganisms adapted to extreme environments. These findings further reinforce that extreme environments remain underexplored sources of bioactive lipid scaffolds and exemplify their potential to inspire new strategies for anti-*T. cruzi* drug discovery.

## 5 Materials and Methods

### 5.1 Sample Collection and Microorganism Isolation

Bacteria isolated from extreme environments were isolated from sediments collected from Torres del Paine National Park, Chilean Patagonia, in collaboration with Universidad de La Frontera, from Bitter Lagoon (50°29′–51°03′S; 72°45′–72°55′W) and Gray Lake (51°03′S; 73°55′W) during the summer season (Figure 7). Samples were maintained under aseptic conditions and transported to the laboratory within 48 h. Bacterial isolation was performed by serial dilution and plating on selective media, using Marine Agar for halophilic bacteria collected from Bitter Lagoon and tryptic soy agar (TSA) for isolates obtained from Gray Lake, followed by incubation at 25 °C for 7 days. Distinct colonies were purified, and bacterial isolates were preserved at −80 °C in cryoprotective medium.

**Figure 7:**
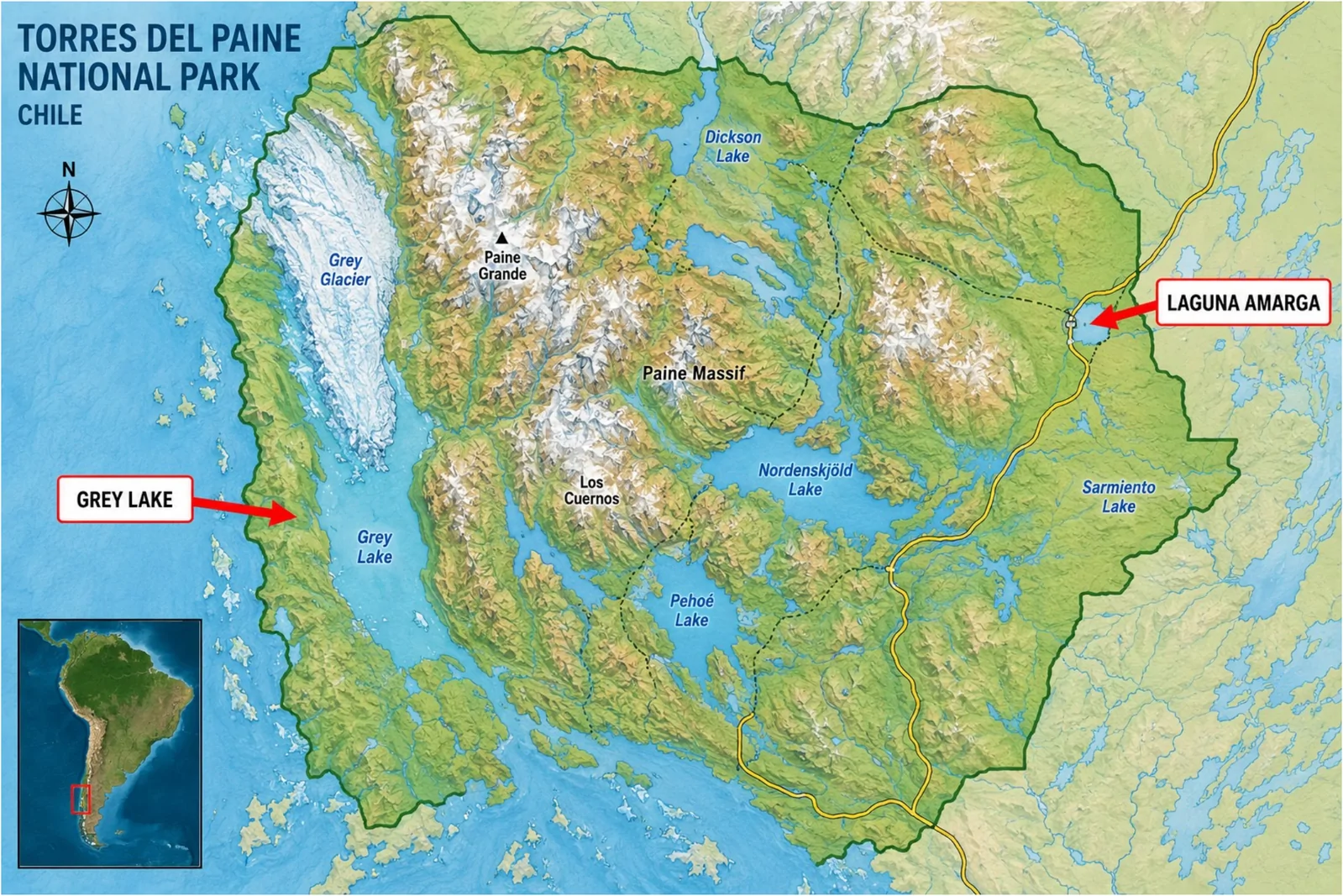
Geographic location of the sampling sites within Torres del Paine National Park, southern Chile. Arrows indicate Bitter Lagoon and Gray Lake, where environmental samples were collected for the isolation of bacteria isolated from extreme environments evaluated in this study. Graphical elements in this figure were generated using OpenAI’s ChatGPT image-generation tool based on scientific instructions provided by the authors and were reviewed and adjusted by the authors for scientific accuracy.

From the bacterial collection established by our group, seven isolates isolated from extreme environments were selected for this study based on differences in colony morphology, growth rate, and stability after subculturing under standard laboratory conditions, including aerobic atmosphere, atmospheric pressure, room temperature, and static incubation. Selected isolates were reactivated on 90 mm × 15 mm Petri dishes containing 10 mL of Marine Agar or TSA and incubated in a BOD incubator at 25 °C for 24 h to monitor growth and colony morphology.^43^

### 5.2 DNA Extraction and 16S rRNA Gene Sequencing of the Bacterial Isolates

Partial 16S rRNA gene sequencing was performed by GoGenetic following the GG-PSC019 workflow. Genomic DNA was extracted from approximately 50 mg of bacterial biomass using the Extracta Pathogen DNA kit (Loccus) according to the manufacturer’s instructions. DNA quantity and purity were determined using a NanoDrop 2000 spectrophotometer (Thermo Fisher Scientific). The 16S rRNA region was amplified in a Veriti thermal cycler (Thermo Fisher Scientific) using 20 ng of genomic DNA and the GoTaq PCR system (Promega). Amplification was performed using the broad-range bacterial primers 27F (5′-AGAGTTTGATCMTGGCTCAG-3′) and 1492R (5′-RGYTACCTTGTTACGACTT-3′).^44^ This primer pair targets the region corresponding approximately to positions 27–1492 of the bacterial 16S rRNA gene and generates an amplicon of approximately 1400 bp. PCR conditions consisted of 32 cycles of denaturation at 96 °C for 30 s, annealing at 60 °C for 45 s, and extension at 72 °C for 90 s. Amplicons were verified by electrophoresis on 1% agarose gels prepared in TAE buffer and subsequently purified using ExoI/SAP treatment (Thermo Fisher Scientific). Purified amplicons were subjected to Sanger sequencing using the BigDye Terminator v3.1 chemistry (Thermo Fisher Scientific). Cycle sequencing was carried out for 35 cycles consisting of denaturation at 96 °C for 15 s, primer annealing at 60 °C for 15 s, and extension at 60 °C for 4 min. Sequencing reactions were precipitated with 20% ammonium acetate (7.5 M) and three volumes of absolute ethanol, resuspended in 10 μL of Hi-Di formamide, and analyzed on a 3500xL Genetic Analyzer (Thermo Fisher Scientific) using 50 cm capillaries filled with POP-7 polymer. Raw chromatograms were processed using Sequencing Analysis v5.4 software (Thermo Fisher Scientific), quality-filtered using a Phred score cutoff >25 and assembled with CAP3 to generate consensus sequences for taxonomic characterization. Consensus sequences were compared with sequences available in the NCBI nonredundant nucleotide database using BLASTn, and isolate identities were assigned based on sequence similarity values using a 99% threshold.^43^

### 5.3 Extraction of Microbial Metabolites

Bacterial isolates were reactivated on three Petri dishes (90 mm × 15 mm) containing 10 mL of Marine Agar or tryptic soy agar (TSA) and incubated in a BOD incubator at 25 °C for 72 h. Subsequently, colonies were subcultured using a 10 μL inoculating loop onto five Petri dishes (150 mm × 15 mm) containing 40 mL of the corresponding culture medium. Plates were incubated at 25 °C for 7 days to allow sufficient biomass production for metabolite extraction. Bacterial biomass was then individually harvested from the agar surface using sterile cell scrapers.

For organic extract preparation, harvested biomass was suspended in 100 mL of chilled ultrapure water (Milli-Q, 4 °C) and subjected to ultrasonic bath sonication for 40 min at room temperature. EtOAc (100 mL) was subsequently added, and the suspension was sonicated for an additional 15 min. The organic phase was filtered using a Büchner funnel and concentrated under reduced pressure at 40 °C. Crude organic extracts obtained from each bacterial species were stored at −20 °C until antiparasitic screening assays were performed.^43^

### 5.4 Bioactivity-Guided Fractionation of *C. plantarum* Extract

To obtain sufficient biomass of *C. plantarum* for fractionation, bacterial cultivation was scaled up using 110 Petri dishes (150 mm × 15 mm) containing Marine Agar. Obtained extract (160 mg) was successively extracted with hexane (3 × 200 mL) and EtOAc (4 × 200 mL). The bioactive EtOAc phase (75 mg) was subjected to solid-phase extraction (SPE) using C_18_ cartridges (Agilent). The cartridges were first conditioned with MeOH, after which the sample was loaded and sequentially eluted with MeOH/H_2_O (1:9), 100% MeOH, and 100% EtOAc, yielding three fractions (F1–F3). All fractions were concentrated under reduced pressure and stored at −20 °C until further analysis.

Fraction F2 (34 mg) displayed antiparasitic activity and were further subjected to HPLC-DAD (Prominence, Shimadzu Co., Japan) using a semipreparative C_18_ column (250 mm × 9.4 mm, 10 μm; Agilent). The initial mobile-phase composition of MeOH:H_2_O 1:9 was maintained for 5 min, followed by a linear increase in MeOH to 100% over 15 min. The mobile phase was then maintained at 100% MeOH for an additional 5 min, after which the column was returned to the initial conditions and re-equilibrated. The flow rate was set at 2.5 mL/min, with an injection volume of 500 μL. This procedure afforded 0.6 mg of compound VAS8 with a purity of 97%, as determined by HPLC.

### 5.5 Mass Spectrometry Analyses

Mass spectra were recorded using an ESI-IT-TOF mass spectrometer (Shimadzu, Kyoto, Japan). Aliquots (0.5 μL) were manually injected through a Rheodyne injector under a constant flow rate of 200 μL min^–1^. The interface voltage, detector voltage, and source temperature were set at 4.5 kV, 1.85 kV, and 200 °C, respectively. Data were acquired in positive ionization mode, and instrument control, data acquisition, and processing were performed using LCMS Solution software (Shimadzu, Kyoto, Japan).^45^

### 5.6 Parasites and Mammalian Cells

*Trypanosoma cruzi* Y strain trypomastigotes were maintained in LLC-MK2 cells cultured in RPMI-1640 medium supplemented with 2% fetal bovine serum (FBS) at 37 °C in a humidified atmosphere containing 5% CO_2_.^46^ Peritoneal macrophages were obtained by washing the peritoneal cavity of BALB/c mice with RPMI-1640 medium supplemented with 10% FBS.

### 5.7 Cytotoxicity Assay

Peritoneal macrophages from BALB/c mice were seeded into 96-well plates at a density of 6 × 10^4^ cells/well and incubated with the VAS8 for 48 h at 37 °C under 5% CO_2_. Compound VAS8 was dissolved in DMSO and serially diluted (150–1.17 μM) in RPMI-1640 medium supplemented with 10% FBS. Cell viability was determined using the MTT assay by adding MTT solution (0.5 mg/mL per well) followed by incubation for 4 h. The reaction was terminated by the addition of 10% SDS, and absorbance was measured after 24 h at 570 nm using a Varioskan LUX Multimode Microplate Reader (Thermo Fisher Scientific).^46^

### 5.8 Antiparasitic Activity Assays

Crude microbial extracts were dissolved in MeOH, diluted in culture medium, and incubated with parasites using a final solvent concentration not exceeding 0.5% (v/v) per well. Compound VAS8 was dissolved in DMSO, diluted in culture medium, and tested under the same solvent limit. The IC_50_ values were determined from three independent biological experiments, each performed in technical duplicate. For each independent experiment, concentration–response data were fitted to a sigmoidal dose–response curve using nonlinear regression in GraphPad Prism version 5.0. The reported IC_50_ values represent the mean ± standard deviation (SD) of the IC_50_ values obtained from the three independent experiments.

For trypomastigote assays, crude extracts were evaluated in 96-well plates at concentrations ranging from 150 to 1.17 μg/mL against *T. cruzi* trypomastigotes (1 × 10^6^ parasites/well) for 24 h at 37 °C under 5% CO_2_. Extract fractions were tested under the same conditions at a fixed concentration of 100 μg/mL, whereas HPLC-derived subfractions obtained during bioactivity-guided fractionation were evaluated at a fixed concentration of 50 μg/mL. The active compound VAS8 was tested at concentrations ranging from 150 to 1.17 μM, and benznidazole was used as the reference drug (150 to 1.17 μM). Trypomastigote viability was assessed by light microscopy based on parasite morphology and motility, as well as by the resazurin fluorimetric assay using a Varioskan LUX Multimode Microplate Reader (Thermo Fisher Scientific) with excitation and emission wavelengths set at 550 and 590 nm, respectively.^46^

For intracellular amastigote assays, peritoneal macrophages from BALB/c mice were seeded into 16-well NUNC chamber slides at a density of 1 × 10^5^ cells/well in RPMI-1640 supplemented with 10% FBS and incubated for 24 h at 37 °C under 5% CO_2_. Cells were then infected with *T. cruzi* trypomastigotes at a parasite-to-macrophage ratio of 10:1 and incubated for 2 h. Noninternalized parasites were removed by washing prior to treatment initiation. Infected macrophages were treated with serial dilutions of the compound VAS8 (100–0.78 μM) prepared in RPMI-1640 supplemented with 2% FBS for 48 h under the same conditions described above.^46^ At the end of the experiment, cells were fixed with methanol and stained with Giemsa. The IC_50_ values for intracellular amastigotes were determined using the infection index, calculated by multiplying the number of infected macrophages by the number of intracellular amastigotes and dividing the result by the total number of macrophages examined. The selectivity index (SI) was subsequently calculated as the ratio between the CC_50_ value in mammalian cells and the IC_50_ value against intracellular amastigotes.^46^

### 5.9 Plasma Membrane Permeability Assay

Alterations in plasma membrane permeability were evaluated using the fluorescent probe SYTOX Green. *T. cruzi* trypomastigotes (2 × 10^6^ parasites/well) were incubated in black flat bottom 96-well plates containing HBSS supplemented with glucose and SYTOX Green (5 μM) under light-protected conditions. Following stabilization of the basal fluorescence signal, parasites were exposed to the compound VAS8 at concentrations corresponding to the previously determined IC_50_ and 2 × −IC_50_ values. Fluorescence was monitored at 5 min intervals for up to 1 h using a Varioskan LUX Multimode Microplate Reader (Thermo Fisher Scientific) with excitation and emission wavelengths set at 504 and 523 nm, respectively. Triton X-100 (0.05%, v/v) was added at the beginning of the experiment as a positive control and at the end to induce complete membrane permeabilization and determine the maximum fluorescence signal. For fluorescence microscopy analyses, trypomastigotes were incubated with SYTOX Green and the isolated compound under the same conditions described above for 30 min. Fluorescence images were acquired using an EVOS M5000 Cell Imaging System (Thermo Fisher Scientific, USA).^46^

### 5.10 Statistical Analysis

IC_50_ and CC_50_ values were determined by nonlinear regression using sigmoidal dose–response curves. Statistical analyses were performed using GraphPad Prism version 5.0 (GraphPad Software, San Diego, CA, USA), and results are presented as mean ± standard deviation (SD). Assays involving crude extracts and the compound VAS8 were conducted in two independent experiments, each performed in technical duplicate. Bioactivity-guided fractionation experiments were carried out at single concentrations in technical duplicate and were used exclusively for activity tracking and selection of active fractions. The SYTOX Green assay was performed in technical triplicate in one representative independent experiment and subsequently confirmed by fluorescence microscopy. Differences between treated groups and the untreated control were analyzed by two-way analysis of variance (ANOVA) followed by Šídák’s multiple comparisons test. A *p* value <0.05 was considered statistically significant.

## Supplementary Material



## Data Availability

The data supporting the findings of this study are available from the corresponding author upon request.

## Notes

16S rRNA gene sequences of marine bacterial isolates; HPLC and MS spectral data of compound VAS8 (PDF)
