# Supplementary material for: Exploring Bacteria from Extreme Environments in Chilean Patagonia for Antitrypanosomal Metabolites
Source: ACS Omega. 2026 Oct 13;11(42):65164–75. doi: 10.1021/acsomega.6c07469 (PMC13717752; doi:10.1021/acsomega.6c07469)
Supplement: Supplementary file 1 [file ao6c07469_si_001.pdf]

Exploring Bacteria from Extreme Environments in Chilean Patagonia for Antitrypanosomal Metabolites

SUPPORTING INFORMATION

Vanessa Albuquerque[1], Julia Maria Godoi Lima[1], Marina de Monroe Gonçalves[2], Jose Aparecido Ferreira de Lima[3], Ravi Kant[4], Myron Christodoulides[5], Daniel Carvalho Pimenta[3], João Henrique Ghilardi Lago[2]*, Cledir Santos[6,7]*, Andre Gustavo Tempone[1]*

[1]Pathophysiology Laboratory, Instituto Butantan, Av. Vital Brazil, 1500, São Paulo 05503-900, SP, Brazil;

[2]Centre of Natural Sciences and Humanities, Federal University of the ABC, São Paulo 09210-580, SP, Brazil;

[3]Laboratory of Ecology & Evolution, Instituto Butantan, Av. Vital Brazil, 1500, São Paulo 05503-900, SP, Brazil;

[4]Computational Drug & Vaccine Discovery Laboratory, Faculty of Applied Sciences & Biotechnology, Shoolini University, Solan Himachal Pradesh-173229, India;

[5]Molecular Microbiology, School of Clinical and Experimental Sciences, Faculty of Medicine, University of Southampton, Southampton SO16 6YD, England-UK;

[6]Scientific and Technological Bioresource Nucleus (BIOREN-UFRO), Universidad de La Frontera, Temuco 4811-230, Chile;

[7]Postgraduate Program in Biotechnology, Federal University of Technology-Paraná, Ponta Grossa 84017220, Brazil.

Supporting Information 1 - 16S rRNA Gene Sequences of Extremophile Isolates

i) *Cyclobacterium plantarum*

TTTCCATGGCTTGACGGGCCCGTGTGTACAAGGTCCGGGAACGTATTCACCGCGCCG

TGGCTGATGCGCGATTACTAGCGATTCCAGCTTCACGGGGCCGGGTTGCAGGCCCCG

ATCCGAACTGAGACGCACTTTTAGAGGTTGGCTTCACGTTGCCGTGTCGCTACCCGC

TGTATGCGCCATTGTAGCACGTGTGTCGCCCCGGGCGTAAGGGCCATGATGACTTGA

CGTCGTCCCCTCCTTCCTCTCCGCTTGCGCGGGCAGTCTTATTAGAGTCCCCAGCTTA

ACCTGATGGCAACTAATAACAGGGGTTGCGCTCGTTGCGGGACTTAACCCAACACCT

CACGGCACGAGCTGACGACAGCCATGCAGCACCTTGTTTTGTGTCCTTGCGGACTGT

TACATCTCTGTAACATTCACTCACATTCTAGCCCGGGTAAGGTTCCTCGCGTATCATC

GAATTAAACCACATGCTCCACCGCTTGTGCGGACCCCCGTCAATTCCTTTGAGTTTC

ACCGTTGCCGGCGTACTCCCCAGGTGGATCACTTAACGCTTTCGCTTGGCCACACAC

GGCTACGCCGTGTACAGCGAGTGATCATCGTTTACGGCGTGGACTACCAGGGTATCT

AATCCTGTTCGCTACCCACGCTTTCGTGCCTCAGCGTCAGTGAACGGCCGGTACAAT

GCCTTCGCTATCGGTGTTCCTTATGGTATCTATGCATTTCACCGCTACACCATAAATT

CCATGTACCCCTCCGTTACTCAAGCCTGACAGTATCAACGGCAGTCTCCCGGTTGAG

CCGGAAACTTTCACCGCTGACTTAACAGGCCGCCTACGCACCCTTTAAACCCAATAA

ATCCGGACAACGCTCGCACCCTCCGTATTACCGCGGCTGCTGGCACGGAGTTAGCCG

GTGCTTATTCATACGGTACCGGCAATCTCCCCCGCAGGGGCGTTTTCTTCCCGTATAA

AAGCAGTTTACAACCCAATAGGCCGTCTTCCTGCACGCGGCATGGCTGGTTCAGGCT

CCCGCCCATTGACCAATATTCCCTACTGCTGCCTCCCGTAGGAGTCTGGCCCGTATCT

CAGTGCCAGTGTGGGGGACCTTCCTCTCAGAACCCCTAAGGATCGTCGCCTTGGTGG

GCCGTTACCTCACCAACTAGCTAATCCTACGCATGCCCGTCTTTCACCGATAAATCTT

TAATATATCAATCATGCAATCAATATATACAATGGGGTATTAATCCGGGTTTCCCCG

GGCTATCCCC

ii) Loktanella agnita

ACGGCCTTCGGGTAAACCCAACTCCCATGGTGTGACGGGCGGTGTGTACAAGGCCC

GGGAACGTATTCACCGCGTCATGCTGTTACGCGATTACTAGCGATTCCGACTTCATG

GGGTCGAGTTGCAGACCCCAATCCGAACTGAGATGCCTTTTGGAGATTAACTCACTG

TAGGCACCATTGTAGCACGTGTGTAGCCCAACCCGTAAGGGCCATGAGGACTTGAC

GTCATCCACACCTTCCTCCCGCTTATCACGGGCAGTTTCTTTAGAGTGCCCAGCCGA

ACTGCTGGCAACTAAAGATGTGGGTTGCGCTCGTTGCCGGACTTAACCGAACATCTC

ACGACACGAGCTGACGACAGCCATGCAGCACCTGTCACTCTGTCTCTTACGAGAAA

AGCCAGTCTCCTGGCCGGTCAGAGGATGTCAAGGGTTGGTAAGGTTCTGCGCGTTGC

TTCGAATTAAACCACATGCTCCACCGCTTGTGCGGGCCCCCGTCAATTCCTTTGAGTT

TTAATCTTGCGACCGTACTCCCCAGGNCNGGAATGCTTAATCCGTTAGGTGTGACAC

CGAAGGGCAAGCCCCCCNGACGTCTGGCATTCATCGTTTACGGGTGTGGACTACCA

GGGNTATCTAATCCTGTTTGCTCCCCACACTTTCGTACCTCAGCGTCAGTATCGAGCC

AGTGAGCCGCCTTNCGCCACTGGTGTTCCTCCAAATATCTACGAATTTCACCTCTAC

ACTTGGAATTCCACTCACCTCTCTCGAACTCTAGACTAGTAGTTTTGGAGGCAGTTCC

AGGGTTGAGCCCTGGGATTTCACCCCCAACTTTCTAATCCGCCTACGTACGCTTTAC

GCCCAGTAATTCCGAACAACGCTAACCCCCTCCGTATTACCGCGGCTGCTGGCACGG

AGTTAGCCGGGGTTTCTTTACCAGGTACTGTCATTATCATCCCTGGCGAAAGAGCTT

TACGACCCTAAGGCCTTCGTCACTCACGCGGCATGGCTAGATCAGGCTTGCGCCCAT

TGTCTAAGATTCCCCACTGCTGCCTCCCGTAGGAGTCTGGGCCGTGTCTCAGTCCCA

GTGTTGCTGATCATCCTCTCAAACCAGCTACTGATCGTAGACTTGGTAGGCCATTAC

CCCACCAACTATCTAATCAGACGCGGGCTAATCCATCACCGATAAATCTTTCCCCCG

AAGGGCGTATACGGTATTACTCATCGTTTCCAATGGCTAT

iii) Pseudarthrobacter humi

GGGGTTAGGCCACCGGCTTCGGGTGTTACCAACTTTCGTGACTTGACGGGCGGTGTG

TACAAGGCCCGGGAACGTATTCACCGCAGCGTTGCTGATCTGCGATTACTAGCGACT

CCGACTTCATGGGGTCGAGTTGCAGACCCCAATCCGAACTGAGACCGGCTTTTTGGG

ATTAGCTCCACCTCACAGTATCGCAACCCTTTGTACCGGCCATTGTAGCATGCGTGA

AGCCCAAGACATAAGGGGCATGATGATTTGACGTCGTCCCCACCTTCCTCCGAGTTG

ACCCCGGCAGTCTCCTATGAGTCCCCGCCATAACGCGCTGGCAACATAGAACGAGG

GTTGCGCTCGTTGCGGGACTTAACCCAACATCTCACGACACGAGCTGACGACAACC

ATGCACCACCTGTAAACCGACCGCAAGCGGGGCACCTGTTTCCAGGTATTTCCGGTT

CATGTCAAGCCTTGGTAAGGTTCTTCGCGTTGCATCGAATTAATCCGCATGCTCCGC

CGCTTGTGCGGGCCCCCGTCAATTCCTTTGAGTTTTAGCCTTGCGGCCGTACTCCCCA

GGCGGGGCACTTAATGCGTTAGCTACGGCGCGGAAAACGTGGAATGTCCCCCACAC

CTAGTGCCCAACGTTTACGGCATGGACTACCAGGGTATCTAATCCTGTTCGCTCCCC

ATGCTTTCGCTCCTCAGCGTCAGTTAATGCCCAGAGACCTGCCTTCGCCATCGGTGTT

CCTCCTGATATCTGCGCATTTCACCGCTACACCAGGAATTCCAGTCTCCCCTACATCA

CTCTAGTCTGCCCGTACCCACCGCAGATCCGGAGTTGAGCCCCGGACTTTCACGGCA

GACGCGACAAACCGCCTACGAGCTCTTTACGCCCAATAATTCCGGATAACGCTTGCG

CCCTACGTATTACCGCGGCTGCTGGCACGTAGTTAGCCGGCGCTTCTTCTGCAGGTA

CCGTCACTTTCGCTTCTTCCCTACTGAAAGAGGTTTACAACCCGAAGGCCGTCATCC

CTCACGCGGCGTCGCTGCATCAGGCTTTCGCCCATTGTGCAATATTCCCCACTGCTG

CCTCCCGTAGGAGTCTGGGCCGTGTCTCAGTCCCAGTGTGGCCGGTCACCCTCTCAG

GCCGGCTACCCGTCGTCGCCTTGGTGAGCCATTACCTCACCAACAAGCTGATAGGCC

GCGAGTCCATCCAAAACCACAATAAAGCTTTCCACCCCCCACCATGCGATGAGGAG

TCATATCCGGTATTAGACCCAGTTTCCCAGGCTTATCCCAGAGTTAAGGGCAGGTTA

CTCACGTGTTACTCACCCGTTCGCCACTAATCCACCCACAAGTGGGCATCAT

iv) Pseudomonas baetica

GGTTAGACTAGCTACTTCTGGTGCAACCCACTCCCATGGTGTGACGGGCGGTGTGTA

CAAGGCCCGGGAACGTATTCACCGTGACATTCTGATTCACGATTACTAGCGATTCCG

ACTTCACGCAGTCGAGTTGCAGACTGCGATCCGGACTACGATCGGTTTTATGGGATT

AGCTCCACCTCGCGGCTTGGCAACCCTTTGTACCGACCATTGTAGCACGTGTGTAGC

CCAGGCCGTAAGGGCCATGATGACTTGACGTCATCCCCACCTTCCTCCGGTTTGTCA

CCGGCAGTCTCCTTAGAGTGCCCACCATTACGTGCTGGTAACTAAGGACAAGGGTTG

CGCTCGTTACGGGACTTAACCCAACATCTCACGACACGAGCTGACGACAGCCATGC

AGCACCTGTCTCAATGTTCCCGAAGGCACCAATCCATCTCTGGAAAGTTCATTGGAT

GTCAAGGCCTGGTAAGGTTCTTCGCGTTGCTTCGAATTAAACCACATGCTCCACCGC

TTGTGCGGGCCCCCGTCAATTCATTTGAGTTTTAACCTTGCGGCCGTACTCCCCAGGC

GGTCAACTTAATGCGTTAGCTGCGCCACTAAGAGCTCAAGGCTCCCAACGGCTAGTT

GACATCGTTTACGGCGTGGACTACCAGGGTATCTAATCCTGTTTGCTCCCCACGCTTT

CGCACCTCAGTGTCAGTATCAGTCCAGGTGGTCGCCTTCGCCACTGGTGTTCCTTCCT

ATATCTACGCATTTCACCGCTACACAGGAAATTCCACCACCCTCTACCATACTCTAG

CTTGTCAGTTTTGAATGCAGTTCCCAGGTTGAGCCCGGGGATTTCACATCCAACTTA

ACAAACCACCTACGCGCGCTTTACGCCCAGTAATTCCGATTAACGCTTGCACCCTCT

GTATTACCGCGGCTGCTGGCACAGAGTTAGCCGGTGCTTATTCTGTCGGTAACGTCA

AAACAGCAAAGTATTAATTTACTGCCCTTCCTCCCAACTTAAAGTGCTTTACAATCC

GAAGACCTTCTTCACACACGCGGCATGGCTGGATCAGGCTTTCGCCCATTGTCCAAT

ATTCCCCACTGCTGCCTCCCGTAGGAGTCTGGACCGTGTCTCAGTTCCAGTGTGACT

GATCATCCTCTCAGACCAGTTACGGATCGTCGCCTTGGTGAGCCATTACCCCACCAA

CTAGCTAATCCGACCTAGGCTCATCTGATAGCGCAAGGCCCGAAGGTCCCCTGCTTT

CTCCCGTAGGACGTATGCGGTATTAGCGTTCCTTTCGAAACGTTGTCCCCCACTACC

AGGCAGATTCCTAGGCATTACTCACCCGTCCGCCGCTGAATTGAGAAGCAAGCTTCT

CTCTTCCGCTCGAC

v) *Psychroflexus aurantiacus*

TGGCTTGACGGGCGGTGTGTACAAGGCCCGGGAACGTATTCACCGCATCATGGCTG

ATATGCGATTACTAGCGATTCCAGCTTCACGCAGTCGAGTTGCAGACTGCGATCCGA

ACTGTGATAGGGTTTGTAGATTCGCTCCTGGTCACCCAGTGGCTGCTCTCTGTCCCTA

CCATTGTAGCACGTGTGTAGCCCAGGACGTAAGGGCCGTGATGATTTGACGTCATCC

CCACCTTCCTCACAGTTTACACTGGCAGTATGGCTAGAGTCCCCATCATTACATGCT

GGTAACTAACCAAAGGGGTTGCGCTCGTTATAGGACTTAACCTGACACCTCACGGCA

CGAGCTGACGACAACCATGCAGCACCTTGCAATTTGCCCGAAGGAAAAGGTGTTTC

CACCCCTGTCAAACTGCATTTAAGCCCTGGTAAGGTTCCTCGCGTATCATCGAATTA

AACCACATGCTCCACCGCTTGTGCGGGCCCCCGTCAATTCCTTTGAGTTTCATTCTTG

CGAACGTACTNCCCCAGGTGGGTTACTTATCACTTTSSSTTAGCCAYYCAGGTCTCAA

ATTAACCCGNAACAGNCTAGTAACCCATCGTTTNACGGGCGTARGACTACCCAGGG

NTATCTNAATCCNTGTTCGCTACCCTACGCTTTCGTCCATCAGGCGTCAGTGTATTGT

TAGTGATCTGCCNTTCGCAATCGGTATTCTGAGTAATATCTATGCATTTCACCGCTAC

ACTACTCATTCTAACCACTTTACAATAACTCAAGAACAACAGTATCAATGGCAATTT

TACGGTTGAGCCGCAAACTTTCACCACTGACTTATCATCCCGCCTACGGACCCTTTA

AACCCAATGATTCCGGATAACGCTTGGATCCTCCGTATTACCGCGGCTGCTGGCACG
GAGTTAGCCGATCCTTATTTTTACAGTACCGTCAACACCCGACACGTCGGGCTTTTTC
TTCCTGTATAAAAGCAGTTTACAACCCATAGGGCAGTCTTCCTGCACGCGGCATGGC
TGGATCAGGATTGCTCCCATTGTCCAATATTCCTCACTGCTGCCTCCCGTAGGAGTCT
GGTCCGTGTCTCAGTACCAGTGTGGGGGATCTCCCTCTCAGGACCCCTATCTATCGTT
GCCATGGTAAGCCGTTACCTTACCATCTAGCTAATAGAACGCATACTCATCTATTAC
CGTAACCTTTAATTATCAAATGATGCCATCCGATAATACCATAAAATATTAATCCAA
ATTTCTCTGGGCTATCTCTTAGTAATAGGTAGATGTATACGCGTTACGCACCCGTGC
GCC

vi) Roseovarius gahaiensis

GTGTACAAGGCCCGGGAACGTATTCACCGCGTCATGCTGTTACGCGATTACTAGCGA
TTCCGACTTCATGGGGTCGAGTTGCAGACCCCAATCCGAACTGAGACAGCTTTTTGG
GATTAACCCATTGTCACTGCCATTGTAGCACGTGTGTAGCCCAACCCGTAAGGGCCA
TGAGGACTTGACGTCATCCACACCTTCCTCCCGCTTATCACGGGCAGTTTCCATAGA
GTGCCCAGCCGAACTGCTGGCAACTAGGGATGTGGGTTGCGCTCGTTGCCGGACTTA
ACCGAACATCTCACGACACGAGCTGACGACAGCCATGCAGCACCTGTCACTGATCC
AGCCGAACTGAAGGAAAGTGTCTCCACTAACCGCGATCAGGATGTCAAGGGTTGGT
AAGGTTCTGCGCGTTGCTTCGAATTAAACCACATGCTCCACCGCTTGTGCGGGCCCC
CGTCAATTCCTTTGAGTTTTAATCTTGCGACCGTACTCCCCAGGCGGAATGCTTAATC

CGTTAGGTGTGTCACCGAACACTACTACTGCCCGACGACTGGCATTCATCGTTTACG

GTGTGGACTACCAGGGTATCTAATCCTGTTTGCTCCCCACACTTTCGCACCTCAGCGT

CAGTATCGAGCCAGTGAGCCGCCTTCGCCACTGGTGTTCCTCCGAATATCTACGAAT

TTCACCTCTACACTCGGAATTCCACTCACCTCTCTCGAACTCTAGACCGATAGTTTTG

GAGGCAGTTCCGGGGTTGAGCCCCGGGATTTCACCCCCAACTTTCCGATCCGCCTAC

GTGCGCTTTACGCCCAGTAATTCCGAACAACGCTAACCCCCTCCGTATTACCGCGGC

TGCTGGCACGGAGTTAGCCGGGGTTTCTTTACCAGGTACTGTCATTATCATCCCTGG

CGAAAGAGCTTTACGACCCTAAGGCCTTCATCACTCACGCGGCATGGCTAGATCAG

GGTTGCCCCCATTGTCTAAGATTCCCCACTGCTGCCTCCCGTAGGAGTCTGGGCCGT

GTCTCAGTCCCAGTGTTGCTGATCATCCTCTAAAACCAGCTATAGATCGTAGGCTTG

GTAGGCCATTACCCCACCAACTACCTAATCTAACGCGGGCCGATCTTTCACCGAAAC

TCTTTCCCCCGAAGGGCGTATACGGTATTAAACCCAGTTTCCCGGGACTATTCCGTA

GTGAAAGGCACGTTCCCACGCGTTACTAACCCGTCCGCCACTAGATCCGAAG

vii) Roseovarius halotolerans

GGTTAGTAACGCGTGGGAACGTGCCTTTCACTACGGAATAGTCCTGGGAAACTGGGT

TTAATACCGTATACGCCCTTCGGGGGAAAGAATTTCGGTGAAAGATCGGCCCGCGTT

AGATTAGGTAGTTGGTGGGGTAATGGCCTACCAAGCCTACGATCTATAGCTGGTTTT

AGAGGATGATCAGCAACACTGGGACTGAGACACGGCCCAGACTCCTACGGGAGGCA

GCAGTGGGGAATCTTGGACAATGGGCGCAAGCCTGATCCAGCCATGCCGCGTGAGT

GATGAAGGCCTTAGGGTCGTAAAGCTCTTTCGCCAGGGATGATAATGACAGTACCTG
GTAAAGAAACCCCGGCTAACTCCGTGCCAGCAGCCGCGGTAATACGGAGGGGGTTA
GCGTTGTTCGGAATTACTGGGCGTAAAGCGCACGTAGGCGGATTGGAAAGTTGGGG
GTGAAATCCCAGGGCTCAACCCTGGAACTGCCTCCAAAACTACCAGTCTAGAGTTCG
AGAGAGGTGAGTGGAATTCCGAGTGTAGAGGTGAAATTCGTAGATATTCGGAGGAA
CACCAGTGGCGAAGGCGGCTCACTGGCTCGATACTGACGCTGAGGTGCGAAAGTGT
GGGGAGCAAACAGGATTAGATACCCTGGTAGTCCACACCGTAAACGATGAATGCCA
GTCGTCGGGTAGCATGCTATTCGGTGACACACCTAACGGATTAAGCATTCCGCCTGG
GGNAGTACGGTCGCAAGATTAAAACTCAAAGGAATTGACGGGGGCCCGCACAAGCG
GTGGAGCATGTGGTTTAATTCGAAGCAACGCGCAGAACCTTACCAACCCTTGACATC
CTGATCGCGGTTACGAGAGATCGTTTCCTTCAGTTCGGCTGGATCAGTGACAGGTGC
TGCATGGCTGTCGTCAGCTCGTGTCGTGAGATGTTCGGTTAAGTCCGGCAACGAGCG
CAACCCACATCCCTAGTTGCCAGCAGTTCGGCTGGGCACTCTATGGAAACTGCCCGT
GATAAGCGGGAGGAAGGTGTGGATGACGTCAAGTCCTCATGGCCCTTACGGGTTGG
GCTACACACGTGCTACAATGGCAGTGACAATGGGTTAATCCCAAAAAACTGTCTCA
GTTCGGATTGGGGTCTGCAACTCGACCCCATGAAGTCGGAATCGCTAGTAATCGCGT
AACAGCATGACGCGGTGAATACGTTCCCGGGCCTTGTACACACCGCCCGTCACACC
ATGGGAGTTGGGTTTACCCGA

Supporting Information 2 – HPLC and MS spectral data of compound VAS8.

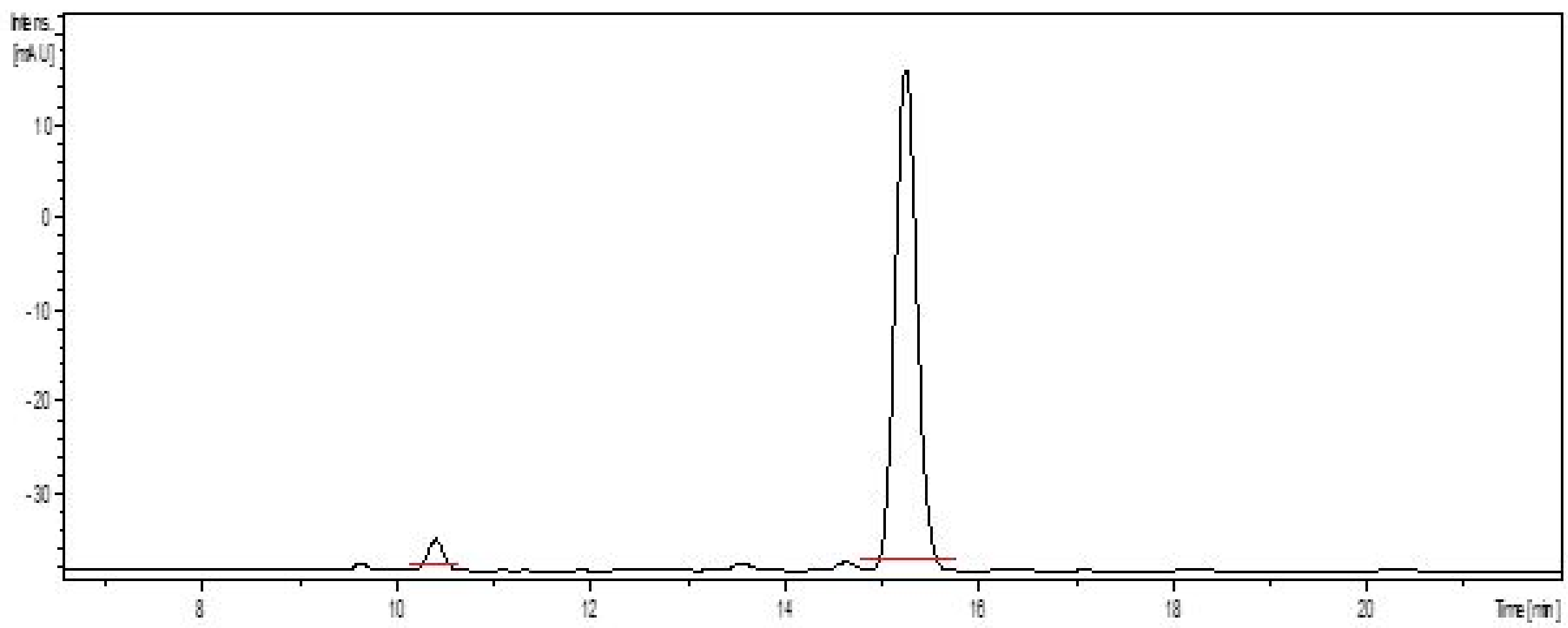

Figure S1 – HPLC analysis of compound VAS8

(C_{18} column, using MeOH:H_2O 1:9 for 5 min increasing the amount of MeOH to 100% during 15 min, then MeOH 100% for 5 min as mobile phase, λ = 220 nm)

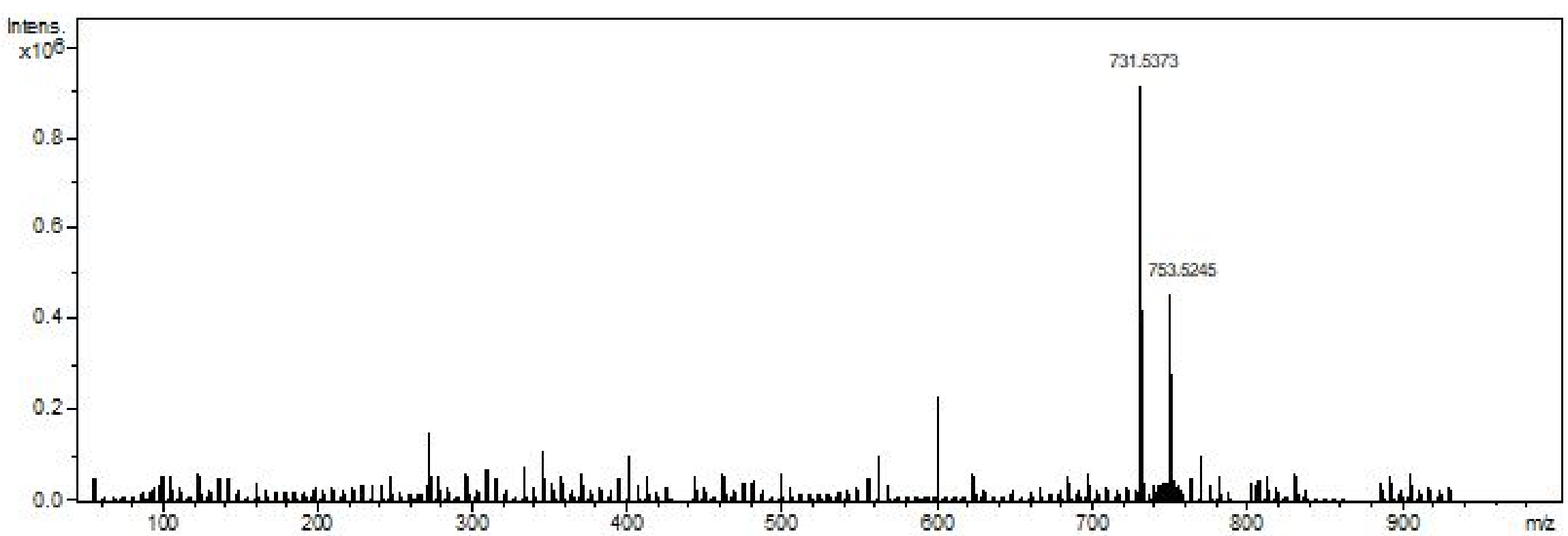

Figure S2 – ESI-HRMS spectrum of compound VAS8 (positive mode).

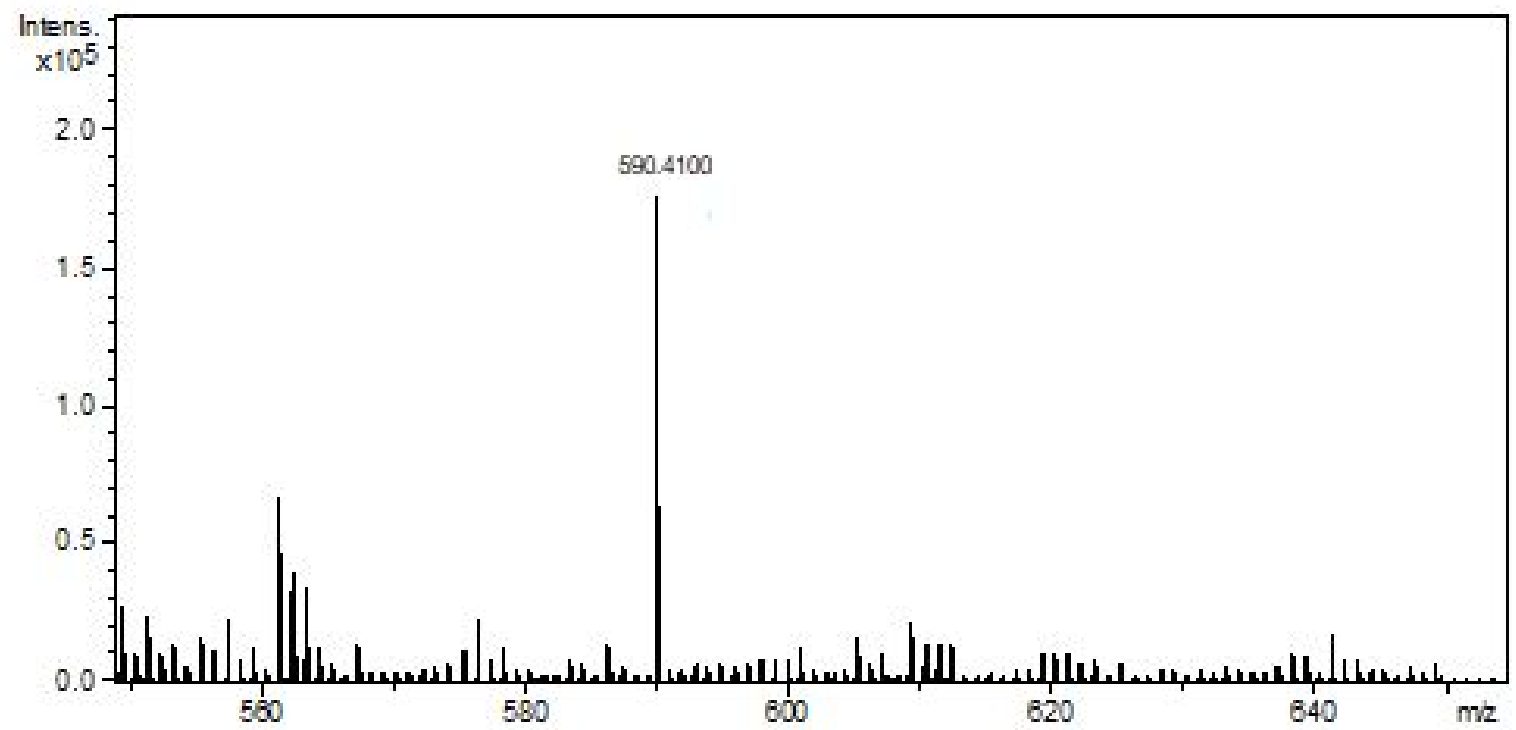

Figure S3 – MS/MS spectrum of compound VAS8 (expansion of *m/z* 550 – 650)

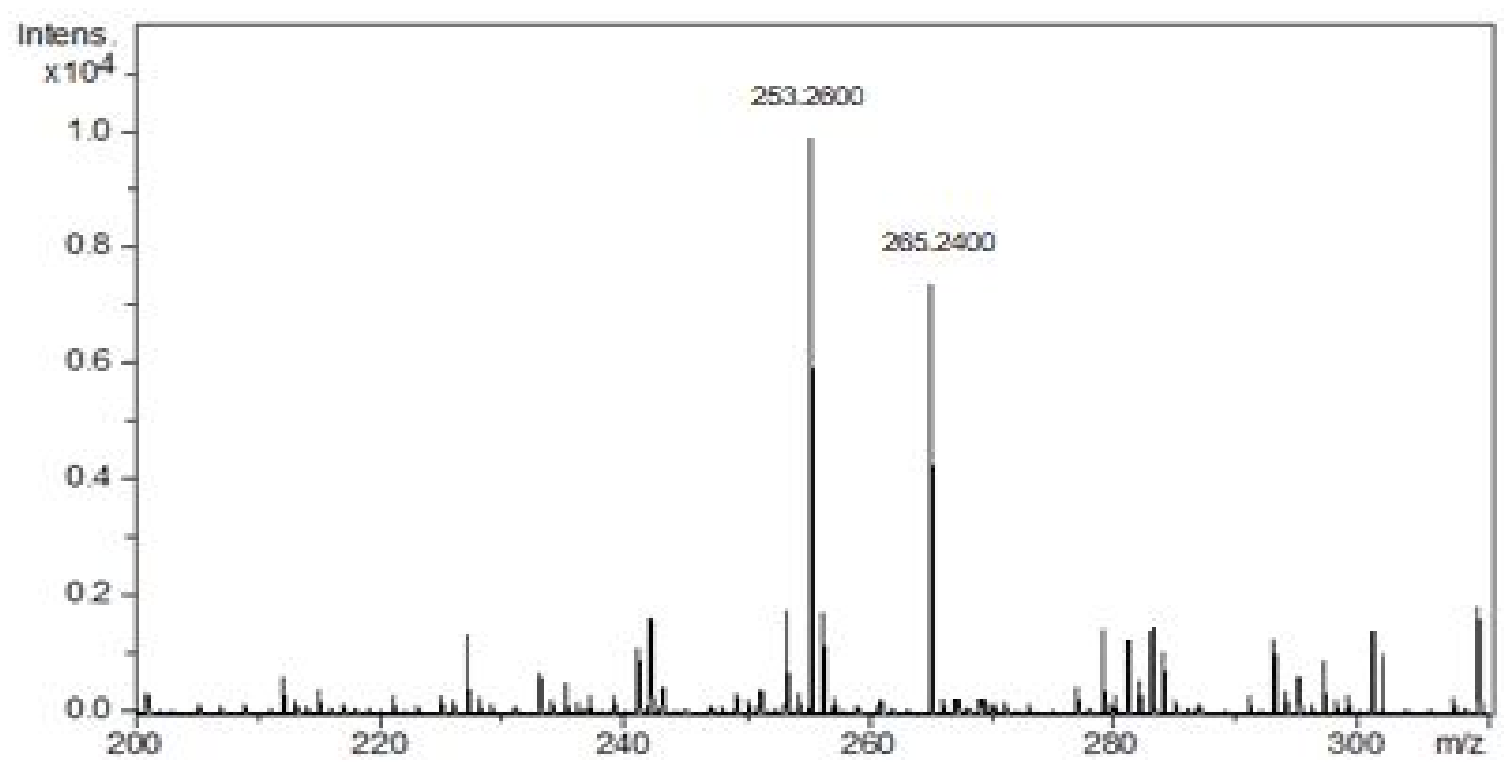

Figure S4 – MS/MS spectrum of compound VAS8 (expansion of *m/z* 200 – 310)

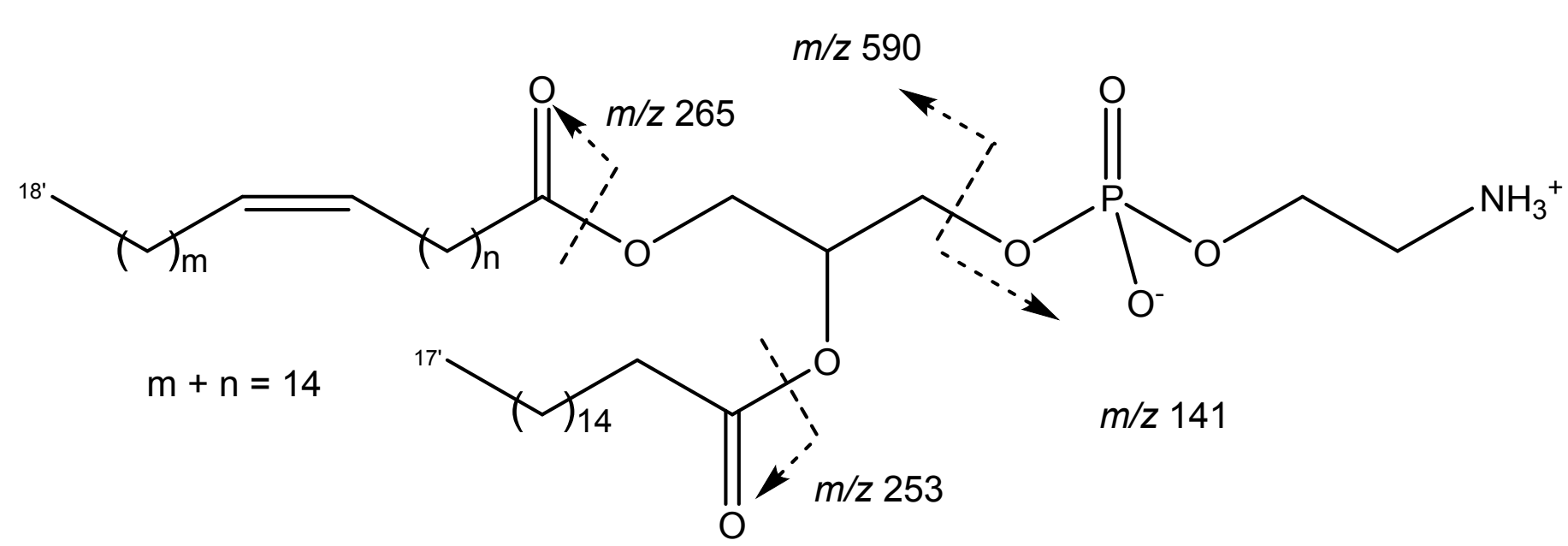

Figure S5 – Fragmentation pattern of compound VAS8
